# 1682. Clinical Characteristics and Outcome of Acinetobacter Bacteremia in Children

**DOI:** 10.1093/ofid/ofad500.1515

**Published:** 2023-11-27

**Authors:** Deema Gashgarey, Mohammed Alsuhaibani, Sami Alhajjar, Ohoud Abdulrahaman Alyabes, Raghad Alhuthil

**Affiliations:** King Faisal Specialist Hospital & Research Centre, Riyadh, Ar Riyad, Saudi Arabia; King Faisal Specialist Hospital & Research Centre, Riyadh, Ar Riyad, Saudi Arabia; King Faisal Specialist Hospital & Research Centre, Riyadh, Ar Riyad, Saudi Arabia; King Faisal Specialist Hospital & Research Centre, Riyadh, Ar Riyad, Saudi Arabia; King Faisal Specialist Hospital & Research Centre, Riyadh, Ar Riyad, Saudi Arabia

## Abstract

**Background:**

*Acinetobacter* is a gram-negative bacterium that can cause several infections, particularly in hospitalized and immunocompromised patients. Invasive *Acinetobacter* infection in pediatrics can lead to significant morbidity and mortality. This study aims to investigate *Acinetobacter* bacteremia clinical characteristics and outcome in pediatric patients.

**Methods:**

This is a retrospective study conducted in a tertiary-care center in Riyadh, Saudi Arabia, including pediatric patients (0-14 years) with a confirmed blood culture of one of the *Acinetobacter* species from January 2015 to December 2022. Medical charts were reviewed for demographics, clinical data, and outcomes. IRB approval number (2231132).

**Results:**

Forty-two pediatric patients with *Acinetobacter* bacteremia were identified. The median age was 10.5 [interquartile range (IQR): 2-48] months, and 25 (59.52%) were males. Over half of the episodes were hospital-acquired infections 26 (61.90%). The most reported subspecies were *Acinetobacter calcoaceticus–Acinetobacter baumannii complex* 34 (81.13%), then *Acinetobacter* lwoffii 4 (9.52%). Polymicrobial infection was found in 15 patients (35.71%), most commonly *Enterococcus faecalis* 4 (26.67). The antimicrobial susceptibility showed that all isolates were susceptible to Colistin, and (68%) to Cefepime and Meropenem. Most of the patients were previously hospitalized 32 (76.19%), exposed to antimicrobial therapy 26 (61.90%), and underwent invasive procedures 23 (54.76%) one month prior to the bacteremia episode. The median duration of treatment was 15 days [IQR: 12-21]. Two patients (4.76%) had relapse (while on treatment), and two (4.76%) had recurrent infection. The overall 14-day crude mortality rate was (11.90%). Interestingly, non-survivors were significantly younger than survivors (median age: 2 [IQR: 1-2] vs. 18 [IQR: 3-48] months respectively.

**Figure:**
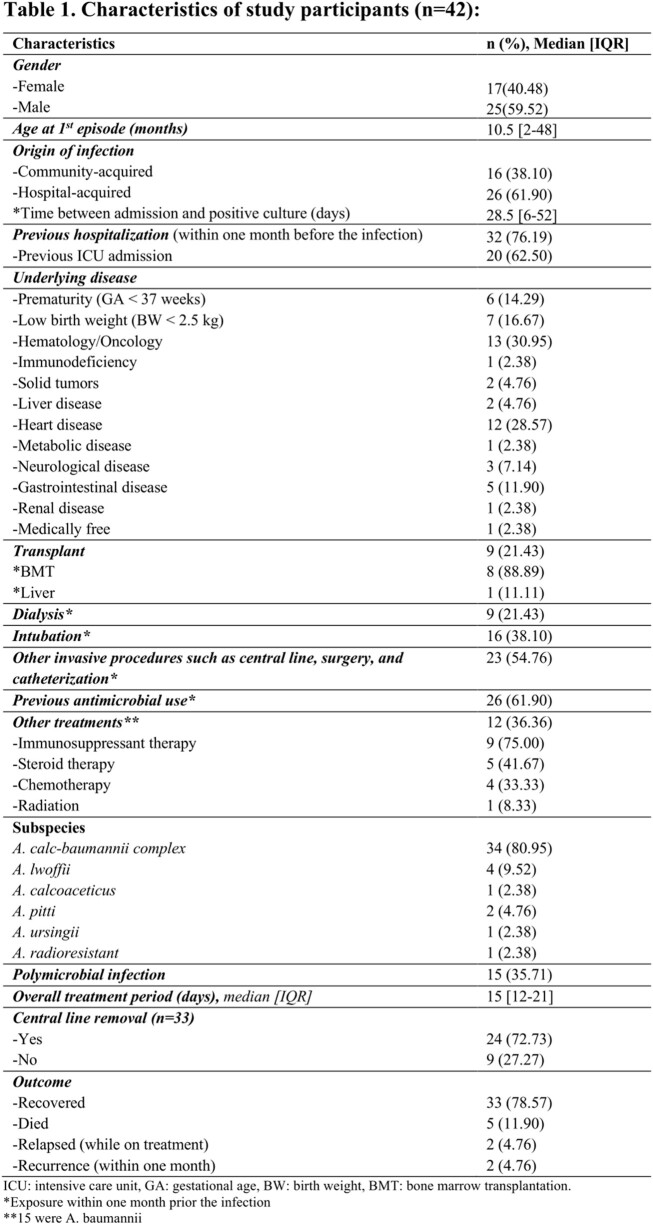

**Figure:**
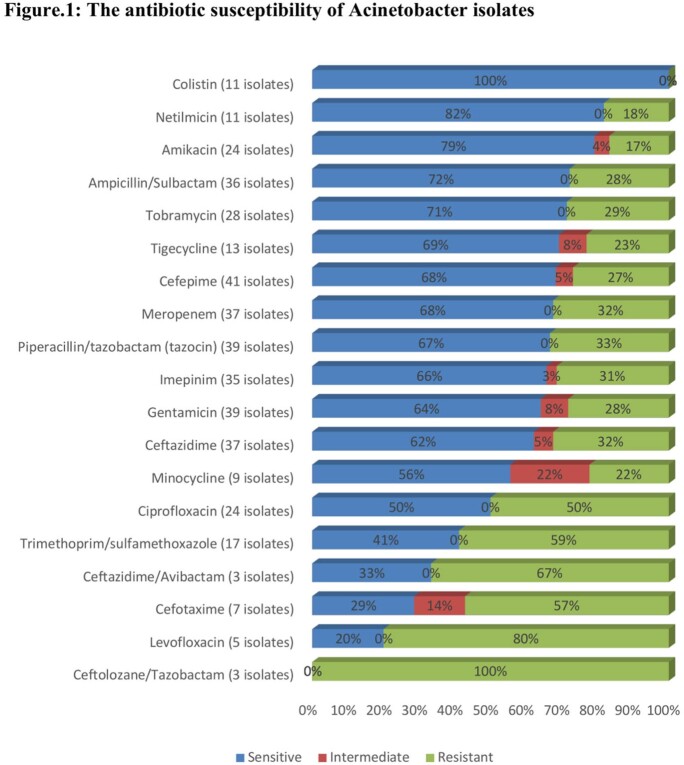

**Conclusion:**

*Acinetobacter* spp. are important emerging nosocomial pathogens. Most of the mortalities were associated with younger age. Previous hospitalization, prior antimicrobial therapy, and invasive procedures were common features for mortality in this study. Further multi-centers studies might be needed to investigate the mortality related risk factors.

**Disclosures:**

**All Authors**: No reported disclosures

